# Tibetan medicine Pa Zhu Wan ameliorates carbon tetrachloride-induced liver fibrosis in rats by regulating the TGF-β-Smad2/3 and IL-6/JAK2/STAT3 signaling pathways

**DOI:** 10.3389/fphar.2025.1667685

**Published:** 2025-11-10

**Authors:** Linlin Zhao, Yan Jiang, Jing Ma, Yongjing Yang, Guo Liu, Zhengxing Wang, Tin Wui Wong, Dejun Zhang

**Affiliations:** 1 Research Center for High Altitude Medicine, Key Laboratory of High-Altitude Medicine (Ministry of Education), Key Laboratory of Application and Foundation for High Altitude Medicine Research in Qinghai Province (Qinghai-Utah Joint Research Key Lab for High Altitude Medicine), Qinghai University, Xining, China; 2 The First People’s Hospital Xining City, Xining, China; 3 College of Eco-Environmental Engineering, Qinghai University, Xining, China; 4 Non-Destructive Biomedical and Pharmaceutical Research Centre, Smart Manufacturing Research Institute, Universiti Teknologi MARA Selangor, Puncak Alam, Selangor, Malaysia; 5 Faculty of Pharmacy, Universiti Teknologi MARA Selangor, Puncak Alam, Selangor, Malaysia

**Keywords:** collagen, inflammation, IL-6/JAK2/STAT3, liver fibrosis, Pa Zhu Wan, TGF-β-Smad2/3

## Abstract

**Background:**

Pa Zhu Wan (PZW) is a Tibetan medicine with natural actives potentially for liver fibrosis treatment. This study determines the therapeutical effects and mechanisms of actions of PZW on carbon tetrachloride-induced hepatic fibrosis in rats.

**Methods:**

The chemical profiles of PZW and its serum metabolites were assessed. The liver elasticity of rats with hepatic fibrosis (induced by carbon tetrachloride-olive oil mixture 1:3 v/v, 0.5 mL/kg) was evaluated against intragastric-fed PZW (0, 60, 120, 240 mg/kg body weight daily for 8 weeks) vs. ursodeoxycholic acid (positive control) with normal rats as negative control by shear wave elastography system (n = 8/group). The serum aminotransferase (ALT and AST), alkaline phosphatase (ALP), bilirubin (TBIL and DBIL), bile acid, inflammatory cytokines (IL-6, IL-1β, TNF-α) and liver fibrosis indicator (HA, LN, PC-III and IV-C) concentrations were measured by ELISA kits. Pathological changes and collagen deposition extent of liver were characterized by immunoassay and H&E/Sirius red/immunohistochemical staining. The expressions of MMP1, TIMP1, IL-6, JAK2, STAT3, p-JAK2, p-STAT3, TGF-β, Smad2/3 and p-Smad2/3 were determined by Western blotting technique.

**Results:**

Piperine, trehalose, mulberroside F, chebulic acid, gallic acid and hydroxysafflor yellow A were main compounds of PZW. PZW alleviated liver fibrosis in a dose-dependent manner with reduced fibrous connective tissue/collagen, pseudolobules and inflammatory cell infiltration. The serum IL-1β, IL-6, TNF-α, TBIL, DBIL, ALT, AST and ALP levels decreased with rats treated with PZW where reduced liver inflammation halted its fibrosis and improved overall hepatic health. PZW mitigated hepatic fibrosis in association with IL-6/JAK2/STAT3 and TGF-β/Smad2/3 signaling pathway inhibition favoring MMP1/TIMP-1 ratio that attenuated collagen deposition and promoted collagen degradation.

**Conclusion:**

PZW alleviated hepatic fibrosis *in vivo*, primarily by inhibiting collagen accumulation through navigating IL-6/JAK2/STAT3 and TGF-β/Smad2/3 signaling pathways.

## 1 Introduction

Liver, as the largest solid organ and gland in human, regulates metabolism, immunity, and secretion physiology (Guilliams et al., 2022). According to epidemiological statistics, hundreds of millions of people around the world currently suffer from chronic liver disease, and about 2 million people die from liver disease each year, of which about 1 million die from cirrhosis or its complications (Santoro and Mangia, 2019). Chronic liver diseases are characterized by progressive pathological phases, which typically start with chronic liver injury, followed by liver fibrosis, and then develop into cirrhosis. Hepatocellular carcinoma (liver cancer) and liver failure are common complications or end-stage outcomes of cirrhosis. Notably, the liver fibrosis stage is reversible. Identification of anti-hepatic fibrosis therapeutics is therefore critical in the treatment of chronic liver diseases.

Liver fibrosis is characterized by phenotypic activation of hepatic stellate cells (HSCs) and excessive secretion of extracellular matrix (ECM) in response to pathogenic factors such as chronic alcoholism, non-alcoholic fatty liver disease, hepatitis viruses, cholestasis, toxins or drugs (Jiang et al., 2024). Liver fibrosis development involves multiple cellular mechanisms where the injured hepatic cells release monocyte chemoattractant protein 1 (MCP1) and transforming growth factor (TGF)-β1 promoting inflammation and activation of HSCs into collagen-producing myofibroblasts (Berumen et al., 2021). In the presence of inflammation, interleukin-6 (IL-6) could activate janus kinase 2 (JAK2)-signal transducer and activator of transcription 3 (STAT3) signaling pathway of which has been reported to lead to liver fibrosis (Ilamathi et al., 2016; Ruan et al., 2021; Schmidt-Arras and Rose-John, 2016). Long-term deposition of ECM by myofibroblasts on the other hand accelerates the progression of liver fibrosis and eventually develops into cirrhosis or hepatocellular carcinoma (Desert et al., 2023). Matrix metalloproteinases (MMPs) and tissue inhibitors of metalloproteinases (TIMPs) are forefront substances of ECM remodeling (Magdaleno et al., 2016). MMPs degrade the ECM whereas TIMPs inhibit ECM degradation, and their imbalance leads to a disturbance in the deposition dynamics of ECM, which promotes liver fibrosis.

Tibetan medicine, with a history of more than 3,800 years, integrates Chinese medicine, ancient Indian medicine and ancient Arabic medicine into a unique medicinal system (Wang et al., 2024). PaMo Zhuba Wan (PMZBW), also known as Pa Zhu Wan (PZW), is a combination of crystalline mirabilite (Gypsum Rubrum: Han Shui Shi), *Chebulae fructus* (*Terminalia chebula Retz*: He Zi), cinnamon (*Cinnamomum tamala (Bauch.-Ham.) Nees et Eberm*: Gui Pi), cardamom (*Amomum roxb*: Dou Kou), pomegranate (*Punica granatum L*: Shi Liu), *Fructus piperis longi* (*Piper longum L*: Bi Ba), pepper (*Piper nigrum L*: Hu Jiao), bright salt (Halite: Guang Ming Yan), Zingiberaceae (*Zingiber striolatum Diel*: Ye Jiang), *Radix aucklandiae* (*Auckiandia lappa decne*: Mu Xiang) and saffron (*Carthamus tinctorius L*: Hong Hua) in a weight ratio of 20∶15∶8∶4∶13∶4∶4∶3∶7∶8∶10 (Supplementary Table S1) (Ministry of Health of the People’s Republic of China, 1995). It is used in Tibetan traditional practice to treat liver disorders as highlighted in eighth century Tibetan medicine classic *Medical Canon in Four Sections* and Pharmacopoeia of the People’s Republic of China (National Pharmacopoeia Committee, 2020). While the pharmacodynamic influences of PZW are known, its pharmacological basis of actions has yet to be explored.

A review of herbal medicine research indicates that various natural therapeutics have been evaluated for their capacity to treat liver fibrosis from the perspectives of anti-viral (helioxanthin, wogonin, glycyrrhizin acid, nobiletin, matrine, oxymatrine, epigallocatechin gallate), anti-inflammatory (genistein, salvianic acid A, botulin, betulinic acid, armepavine, rhein, ginseng, osthole), anti-oxidant (silybin, isosilybin, silydianin, silychristin, curcumin, β-caryophyllene, osthole), apoptotic (silymarin), cytokine inhibitory (rosmarinic acid, baicalin, paeoniflorin, oleanolic acid, ursolic acid, obeticholic acid, ginseng), anti-fibrotic (plumbagin) and epithelial-mesenchymal transition/mesenchymal-epithelial transition modulatory (salvianolic acid B, Diwu Yanggan) activities (Dai et al., 2022; Li et al., 2020; Peng et al., 2023; Wang S. J. et al., 2023; Xiao et al., 2020). PZW is constituted of natural products that can potentially act on the progress of liver fibrosis through a collective pharmacological action of its active principles. Our preliminary pre-clinical trials demonstrated that PZW can increase the peroxisome proliferator-activated receptor (PPAR) expression in the liver tissue which suggested its potential to ameliorate oxidative stress essential to curb liver fibrosis. As such, this study aims to determine the therapeutical effects of PZW on carbon tetrachloride-induced hepatic fibrosis in rats and to investigate its mechanisms of actions in order to provide a theoretical basis for the clinical use of PZW in liver fibrosis treatment. The roles of JAK2/STAT3 and TGF-β/Smad2/3 regulatory pathways are examined specifically as it is found to be a crucial pathogenic mediator of liver fibrosis (Hu et al., 2018; Zhang et al., 2022).

## 2 Materials and methods

### 2.1 Materials

PZW (product registration number: Z20200834000) was provided by Qinghai Tibetan Hospital, Qinghai, China. Ursodeoxycholic acid (UA; H20181,059, Losan Pharma GmbH, Germany) as positive control was supplied by Xining First People’s Hospital, Qinghai, China. Carbon tetrachloride (Titan Technology Shanghai Co. Ltd., China) was used to induce liver fibrosis with olive oil (Haoan Biotechnology Co. Ltd., China) as its carrier. Alanine aminotransferase (ALT; C009-2-1), aspartate aminotransferase (AST; C010-2-1), alkaline phosphatase (ALP; A059-2-2), total bile acid (TBA; E003-2-1), total bilirubin (TBIL; C019-1-1), and direct bilirubin (DBIL; C019-2-1) kits (Nanjing Jiancheng Bioengineering Institute., China) were used to test liver function indices. The enzyme-linked immunosorbent test (ELISA) kits for interleukin 1 beta (IL-1β; E-EL-M0037), interleukin 6 (IL-6; E-EL-M0044), tumor necrosis factor-α (TNF-α; E-EL-M3063) (Elabscience Biotechnology Co. Ltd., China), hyaluronic acid (HA; JL12953), laminin (LN; JL20286), type III procollagen (PC-III; JL20799) and type IV collagen (IV-C; JL20800) (Jianglai Biotechnology Co. Ltd., China) were employed to examine the state of liver inflammation and fibrosis. Sirius Red (G1472; Solarbio Life Sciences, China) was used as the staining solution for collagen. Hematoxylin (G1004; Wuhan Servicebio Technology Co. Ltd., China) and eosin (R34134; Bomei Biotechnology Co. Ltd., China) were used in histological staining. Horseradish peroxidase (HRP) secondary antibody (GB23303; Wuhan Servicebio Technology Co. Ltd., China), alpha-smooth muscle action (α-SMA; ab5694; Abcam Co. Ltd., China), collagen I (14695-1-AP; Proteintech, China) antibodies and diaminobenzidine (DAB; Wanleibio, China) were used in immunohistochemistry study. HRP-goat anti-rabbit immunoglobulin G (IgG; AS014), β-actin (AC038), MMP1 (A22080), TIMP1 (Ab131327), α-SMA (A2235), IL-6 (A0286), JAK2 (A7694), p-JAK2 (AP0531), STAT3 (A1192), p-STAT3 (AP0070), TGF-β (A2124), Smad2/3 (A7536), p-Smad2/3 (A7248) antibodies (ABclonal Biotechnology Co. Ltd., China) were adopted in molecular biology study.

### 2.2 Experimental animals

A total of 60 specific pathogen-free male *Sprague Dawley* rats (200 ± 20 g body weight/rat) were obtained from Beijing Hua Fu Kang Biological Science and Technology Co., China (license no.: SCXK (Beijing) 2019-0008; animal quarantine certificate no.: 1101078403) for serum chemical profiling and liver fibrosis analysis. These rats were fed with pelleted food (Jiangsu Xietong Pharmaceutical Bio-engineering Co. Ltd., China) and had water *ad libitum*. They were kept in ventilated cages at 22 °C–24 °C and 45%–50% relative humidity with a 12-h dark/light cycle. The rats were subjected to at least 7 days of acclimatization. Prior to experiments, all rats were subjected to fasting for at least 8 h. The animal experimentation was carried out in compliance with the ethics regulations of the Medical College of Qinghai University (project license no.: 2022-21) approved on 02 March 2022, meeting the criteria of the Chinese Animal Protection Act and the National Research Council. The overall workflow diagram was presented as Supplementary Figure S1.

### 2.3 PZW chemical profiling

Supplementary Table S1 summarized the composition of PZW where the botanical drugs were collected from the Tibetan plateau region of China and subjected to formulation in September 2023. The botanical drugs were authenticated by Professor Wang Jun from Qinghai Institute for Drug Control and Inspection, China, and the specimen were kept in the herbarium of Qinghai Institute for Drug Control and Inspection, China. PZW was composed of a relative proportion of 200 mg crystalline mirabilite mineral, 150 mg *Chebulae Fructus* seed, 80 mg cinnamon bark, 40 mg cardamom fruit, 130 mg pomegranate pericarp, 40 mg *Fructus Piperis Longi* fruit, 40 mg pepper fruit, 30 mg bright salt, 70 mg ginger root, 80 mg *R. aucklandiae* root and 100 mg saffron flower.

PZW was first ground into powder in a liquid nitrogen. One mL of deionized water was added to 100 mg of ground powder in 1.5-mL centrifuge tube. The mixture was subjected to vortex for 1 min, added with steel beads and pre-cooled at –40 °C for 2 min followed by bead milling at 60 Hz for 2 min to further reduce the particle size of powder for aqueous extraction. The extraction continued by means of ice bath-sonication for 60 min. The extract in the form of a supernatant was collected by centrifugation (Shanghai Lu Xiangyi Centrifuge Instrument Co. Ltd., China) at 14,000 rpm and 4 °C for 10 min. The yield of the hot air-dried extract mass was approximately 17%. The chemical profiles of PZW extract, diluted 10-fold with deionized water, were analyzed with an ACQUITY UPLC I-Class HF ultra-high performance liquid chromatography tandem QE high-resolution mass spectrometer system (Waters, United States). The UHPLC was fitted with an ACQUITY UPLC HSS T3 column (100 mm × 2.1 mm, 1.8 μm; Waters, United States) and a photodiode array detector. Water and acetonitrile (Thermo Fisher Scientific, United States) made up the mobile phase A and phase B respectively. A gradient elution was adopted in chromatography separation: 0 min: 5% B, 95% A; 2 min: 5% B, 95% A; 4 min: 30% B, 70% A; 8 min: 50% B, 50% A; 10 min: 80% B, 20% A; 14 min: 100% B, 0% A; 15 min: 100% B, 0% A; 16 min: 5% B, 95% A. The mobile phase flow rate was maintained at 0.35 mL/min. The sample volume was 5 μL. The photodiode array wavelength scanning range was 210–400 nm. The column temperature was set at 45 °C.

Thermo-Obritrap-QE system (Thermo Fisher Scientific, United States) was used in mass spectrometric analysis involving negative and positive ion modes. The following operating parameters were applied: spray voltage = −3000 V (negative) and 3,800 (positive), capillary temperature = 320 °C, auxiliary gas heater temperature = 350 °C, sheath gas flow rate = 35 arb, auxiliary gas flow rate = 8 arb, S-lens radiofrequency level = 50, mass range = 100–1,200 (m/z), full MS resolution = 70,000, MS/MS resolution = 17,500, normalized collision energy (NCE)/stepped NCE = 10, 20, 40. Baseline filtering, peak identification, integration, retention time correction, peak alignment and normalization of data were performed using Progenesis QI v3.0 software (Nonlinear Dynamics, United Kingdom). The plant component database contains information on over 5,000 component standards (standards supplied by Chengdu Lemeitian Pharmaceutical Technology Co. Ltd., Shanghai Yuanye Biotechnology Co. Ltd. *etc.*), covering 10 major categories of components such as alkaloids, phenolic acids, flavonoids, coumarins, phenylpropanoids, lignans, terpenoids and others.

### 2.4 Serum chemical profiling

Twelve *Sprague Dawley* rats were orally administered with PZW at 240 mg/kg twice daily for 7 consecutive days. At day 8, blood (3–5 mL) was sampled from the abdominal aorta of each rat through abdominal dissection following anesthesia by means of phenobarbital sodium (Tianjin Jinyao Pharmaceutical Co. Ltd., China) at a dose of 0.04 g/kg and all blood samples were pooled. One hundred μL of blood was added with 400 μL of protein precipitant methanol-acetonitrile (2∶1 v/v; containing L-2-chlorophenylalanine 2 μg/mL). The sample was vortexed for 1 min (Shanghai Hanuo Instrument Co. Ltd., China), ultrasonicated for 10 min in an ice-water bath (Shenzhen Fuyang Technology Group Co. Ltd., China), and centrifuged at 4 °C and 12,000 rpm for 10 min. The supernatant was filtered through a 0.22 μm microfilter (Merck, Germany) and stored in vials at −80 °C until liquid chromatography-mass spectrometry (LC-MS) analysis.

The serum sample was thawed at 56 °C for 30 min using a thermostated water bath (YiHeng, ShangHaiYiHeng, China). A Dionex Ultimate 3000 RS UHPLC fitted with Q-Exactive plus quadrupole-Orbitrap mass spectrometer equipped with heated electrospray ionization (ESI) source (Thermo Fisher Scientific, United States) was used to profile the metabolites in both ESI positive and ESI negative ion modes. An ACQUITY UHPLC HSS T3 column (1.8 μm, 2.1 mm × 100 mm; Waters, United States) was employed as the chromatography column. The binary gradient elution system consisted of (A) water (containing 0.1% formic acid) and (B) acetonitrile (containing 0.1% formic acid). The following chromatographic gradient was adopted: 0 min: 5% B, 95% A; 2 min: 5% B, 95% A; 4 min: 25% B, 75% A; 8 min: 50% B, 50% A; 10 min: 80% B, 20% A; 14 min: 100% B; 15 min: 100% B; 16 min: 5% B, 95% A. The flow rate was kept at 0.35 mL/min and the column temperature was 45 °C. The sample injection volume was 3 μL. The mass spectrometry analysis was operated using the following parameter: spray voltage = +3800 V (positive) and = 3000 V (negative), sheath gas flow rate = 35 arb, auxiliary gas flow rate = 8 arb, capillary temperature = 320 °C, auxiliary gas temperature = 350 °C, S-lens radiofrequency level = 50, mass range = 100-1,000 (m/z), full MS resolution = 70,000, MS/MS resolution = 17,500, collision energy = 10, 20 and 40 eV.

### 2.5 *In vivo* study and liver fibrosis analysis

The rats were divided into 6 groups (n = 8/group): normal group (CON) and rats with liver fibrosis classified as negative control group (MOD), positive control group treating with ursodeoxycholic acid at 90 mg/kg (UA), and groups receiving PZW at 60 mg/kg (PZWL), 120 mg/kg (PZWM) and 240 mg/kg (PZWH) *via* oral gavage in 2 mL of physiological saline daily for 8 weeks. The animal doses were obtained through the conversion of the human dose considering differences in body surface area between human and rat (Blanchard and Smoliga, 2015; Nair et al., 2018), where body surface area = k × body weight^0.67^ and k_rat_/k_human_ is the conversion factor. The equivalent human-to-rat converted dose was 120 mg/kg. Low dose was defined as half of the equivalent dose and high dose was set at twice of the equivalent dose. Ursodeoxycholic acid was used as the positive control as it negates liver fibrosis *via* suppressing the TGF-β/Smad signaling pathway of which represents a critical route to collagen deposition and could activate 5′adenosine monophosphate-activated protein kinase (AMPK) pathway in association with JAK/STAT3 inhibition (Liang et al., 2009; Ye et al., 2020; Zhao et al., 2021). Ursodeoxycholic acid stabilizes mitochondrial membrane, reduces reactive oxygen species production and inhibits lipid peroxidation of which directly counteracts the primary mechanism of carbon tetrachloride in hepatocyte damage (Nava-Ocampo et al., 1997; Mas et al., 2008). It suppresses the activation of NF-κB pathway and reduces the secretion of pro-inflammatory cytokines, mitigating the inflammatory drive of fibrosis (Peng et al., 2024). Ursodeoxycholic acid could downregulate α-SMA and collagen expression and promote ECM degradation by upregulating MMPs, to antagonize the fibrotic process (El-Gendy et al., 2021). In carbon tetrachloride-induced liver fibrosis rodent, it has been reported to downregulate fibrotic genes (COL1A1, TGF-β1) (Pirinççioğlu et al., 2012). Except for the normal group, hepatic fibrosis was first induced by intraperitoneally injecting the rats with carbon tetrachloride-olive oil mixture (1∶3 v/v, 0.5 mL/kg) twice a week for 8 weeks at 8–9 a.m. on Wednesday and 8–9 p.m. on Sunday (Kang et al., 2016). The development of liver fibrosis was confirmed by color Doppler ultrasound (Shenzhen Mindary BioMedical Electronics Co. Ltd., China).

For the purpose of animal liver characterization, hepatic ultrasound examination was performed on the abdominal area of the rats at week 8 post-treatment using a two-dimensional shear wave elastography system of which is constituted of an ultrasound diagnostic apparatus (Shenzhen Mindary BioMedical Electronics Co. Ltd., China) equipped with a SL15-4 transducer. The ultrasound operation was set to the superficial (thyroid) imaging mode in accordance with a modified method of Gu et al. (2021). The rats were fasted overnight prior to shear wave elastography examination. They were anesthetized using 2.6% sevoflurane (Lunan Better Pharmaceutical Co. Ltd., China) as a 1∶2 mixture with O_2_ (Peng et al., 2020). The anesthetized rats were placed in supine position and had their fur shaved to fully expose their upper abdomen. A thin layer of ultrasound transmission gel (Shanghai SNWI Medical Co. Ltd., China) was evenly spread on the abdominal skin followed by contact-mode doppler ultrasonic scanning to obtain the 2D ultrasonogram image of liver. The shear wave elastography sampling frame was set at 10 mm × 10 mm and was taken 1–2 mm below the liver capsule at an ultrasound pressure of 0–50 kPa. A region of interest (5 mm diameter), with color accounted for more than 90% of the sampling frame, image which was stable for 3 to 4 s and motion stability index reaching 3 stars or above, was subjected to quantitative measurement to provide the mean modulus of elasticity in kPa. A higher mean modulus of elasticity value denotes greater liver stiffness and a higher extent of hepatic fibrosis. Five liver stiffness measurements were performed in each rat and the average liver elasticity was calculated.

Where applicable, all samples were administered by oral gavage once a day for 8 weeks following the development of liver fibrosis (Chen et al., 2022). Throughout the 8-week duration, the behavior, food intake, body weight, fur color, heart rate and respiration pattern were monitored once weekly. Both food intake and body weight were characterized by gravimetric measures. The heart rate and respiratory rate were determined by small animal pulse oximeter (MouseOx^®^, Shanghai Yuyan Instruments Co. Ltd., China). At day 1 of week 9, all rats were anesthetized by means of phenobarbital sodium at a dose of 0.04 g/kg. The rats were subjected to hepatic ultrasound examination. They were then dissected at abdominal cavity with scalpels and scissors. Blood samples were collected from the abdominal aorta and centrifuged at 3,500 rpm for 10 min at 4 °C to obtain the serum for biochemical and metabolic analysis. The livers were removed, weighed and aliquoted with a part of the liver tissue being stored in 10% formalin solution for histology study, while the rest of the liver tissue was immediately frozen in liquid nitrogen at −80 °C before proceeding to Western blot analysis.

### 2.6 Serum biochemical and liver inflammation/fibrosis marker analysis

The serum TBIL, DBIL, TBA, ALT, AST and ALP levels were assessed using the biochemical diagnostic kits (Nanjing JianCheng Bioengineering Institute Co. Ltd., China) in examination of hepatic health. Serum levels of the inflammatory factors IL-1β, IL-6 and TNF-α were detected using ELISA kits (Elabscience Biotechnology Co. Ltd., China). Serum levels of liver fibrosis markers (HA, LN, PC-III, IV-C) were detected by ELISA kits (Elabscience Biotechnology Co. Ltd., China). Optical density was measured according to the kits’ instructions and serum marker levels were calculated from the standard calibration plots.

### 2.7 Histopathological and immunohistochemistry analysis

The right lobe of the liver tissue was sectioned using scalpel blade (Jinzhong Surgical Instrument, China) and fixed in 10% formalin at 25 °C for 24 h. It was embedded in paraffin (Shanghai Hualing Healing Equipment Factory, China), cut into 4 μm-thick sections using a rotary slicer (Leica Biosystems, Germany), and stained with hematoxylin and eosin (H&E) to assess the histopathological degree of liver injury. Alternatively, the 4 μm-thick sections were stained with Sirius Red in identification of collagen deposit in liver.

Immunohistochemistry staining was performed on the paraffin sections of liver. Liver sections were deparaffinized with dimethylbenzene (Tianjin Zhiyuan Chemical Reagent Co. Ltd., China), polarized with different concentrations of alcohol (100, 85, 75%), and washed with deionized water 3 times. The sections were then heated in citrate buffer (pH 6.0) for antigen retrieval. Endogenous peroxidase activity was blocked with 3% hydrogen peroxide (Sinopharm Chemical Reagent Co. Ltd., China) at room temperature for 10 min and washed with phosphate buffer saline 3 times. The sections were blocked with bovine serum for 20 min and incubated with primary antibody against α-SMA (1∶200) and collagen I (1∶1,000) at 4 °C overnight. HRP labelled secondary antibody goat anti-rabbit IgG (1∶100) was subsequently introduced, incubated at room temperature for 30 min and washed with phosphate buffer saline 3 times, followed by DAB coloration and hematoxylin re-staining. The digital microscope camera system (Motic China Group Co. Ltd., Xiamen, China) was used to capture images of each part, and Halo software (Indica Labs, United States) was used for analysis.

### 2.8 Western blotting

The total protein was extracted from the hepatic tissues which was stored at −80 °C, and protease and phosphatase inhibitors were added to protect the protein and phosphorylated proteins from degradation. The protein concentration was determined by bicinchoninic acid (BCA) method using a colorimetric assay. The total protein (30 μg) was electrophoresed on sodium dodecyl sulfate-polyacrylamide gel electrophoresis (SDS-PAGE) gel. Proteins, separated by SDS-PAGE gel electrophoresis, were transferred to nitrocellulose filter (NC; ABclonal Technology Co. Ltd., China) under a current of 400 mA and at 4 °C for approximately 30 min. NC membrane was then blocked with blocking buffer (Beyotime Biotech Inc., China) for 2 h at 37 °C and incubated with primary antibodies against α-SMA (1:500), collagen I (1:500), MMP1 (1:2,000), TIMP1 (1:1,000), IL-6 (1:1,000), JAK2 (1:1,000); p-JAK2 (1:500), STAT3 (1:1,000), p-STAT3 (1:500), TGF-β (1:1,000), Smad2/3 (1:500), p-Smad2/3 (1:500) and β-actin (1:50,000) at 4 °C overnight. It was then washed with tris-buffered saline buffer (TBST) three times. The NC membrane was subsequently incubated with HRP-labelled goat anti-rabbit secondary antibody for 1 h at room temperature and washed with TBST three times. The membrane was visualized using an enhanced chemiluminescence kit (ABclonal Technology Co. Ltd., China) and Odyssey Infrared Imaging system (LICOR Bioscience, United States). The target protein was quantitatively analyzed by ImageJ software (National Institutes of Health, United States) with β-actin as the internal reference.

### 2.9 Statistical analysis

All data were presented as mean ± standard deviation. Statistical analysis was performed using SPSS 26 (SPSS Science Inc., United States). One-way analysis of variance (ANOVA) was adopted with Tukey’s HSD test applied to all ANOVA-based analyses unless otherwise stated. A *p* value <0.05 was denoted as statistically significant.

## 3 Results and discussion

### 3.1 Chemical profiling

Using UHPLC-MS technique, more than 1,000 compounds were identified in the PZW extract. Piperine (32.8%; parent ion (m/z): 286.1437; product ion (m/z): 135.0441, 201.0547, 286.1438), trehalose (12.6%; parent ion (m/z): 365.1057; product ion (m/z): 203.0525, 365.1053), mulberroside F (11.3%; parent ion (m/z): 611.1635; product ion (m/z): 491.1197, 611.1635), chebulic acid (7.8%; parent ion (m/z): 355.0309; product ion (m/z): 111.0087, 161.0609, 163.0401, 179.0717, 193.0145, 205.0507, 249.0411, 337.0204, 355.0313), gallic acid (7.6%; parent ion (m/z): 339.0359; product ion (m/z): 58.438, 65.2208, 65.2405, 67.775, 70.4296, 70.4524, 76.3228, 125.0244, 169.0144, 206.2047) and hydroxysafflor yellow A (6.8%; parent ion (m/z): 613.1771; product ion (m/z): 313.0708, 331.0811, 355.0811, 367.0809, 397.0915, 415.1022, 433.1130, 451.1234, 595.1648, 613.1765) were the main compounds of the PZW extract (Supplementary Figure S2).

Ursodeoxycholic acid, taurodeoxycholic acid and taurocholic acid have been found to be able to alleviate liver injury *via* anti-oxidant, apoptotic and anti-fibrotic mechanisms (Wang D. et al., 2023; Xu et al., 2018; Yang et al., 2023). Preliminary trials in our laboratory indicated that the ursodeoxycholic acid, taurodeoxycholic acid and taurocholic acid can mitigate the development of carbon tetrachloride-induced liver fibrosis *in vivo*. Oral ingestion of PZW by rats was associated with the formation of metabolites such as taurodeoxycholic acid, 9-hydroxyoctadecanoylcarnitine, docosapentaenoic acid, 4-methylumbelliferone glucuronide and others as detected from the serum by means of LC-MS assay. The findings indicated that both PZW chemical compounds and their metabolites could contribute to liver injury amelioration. On this note, the therapeutic potential of PZW against hepatic fibrosis was investigated in the subsequent study using ursodeoxycholic acid as the positive control.

### 3.2 Liver stiffness

**FIGURE 1:**
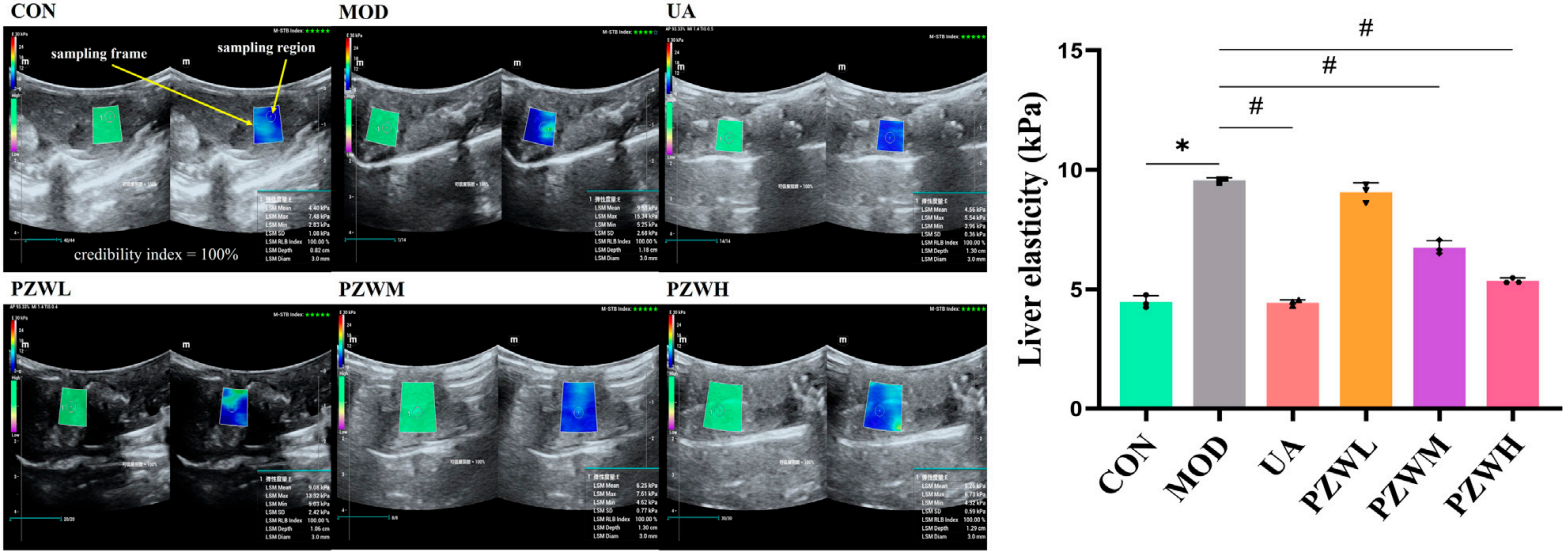
The liver ultrasonogram and elasticity profiles of normal rats, rats with carbon tetrachloride-induced liver fibrosis and those treated with UA and PZW. The elasticity value was expressed as mean ± standard deviation. * *p* < 0.05 against CON, # *p* < 0.05 against MOD.

Unlike CON, the MOD group was characterized by a higher level of liver stiffness as indicated by the shear wave elastography measurement (Figure 1). They had reduced food intake (from 1,668.8 ± 12.7 g/group to 422.3 ± 15.3 g/group) and body weight (from 199.9 ± 1.2 g/rat to 146.8 ± 0.7 g/rat). They were physically less active with yellowish dull fur and had increased respiration rate (from 88.38 ± 2.37 breath/min to 134.50 ± 1.01 breath/min) and heart rate (from 435.20 ± 1.97 beat/min to 501.78 ± 3.75 beat/min). Treatment of these rats with PZW generally reduced their liver stiffness in the following dose progression: PZWH < PZWM < PZWL (Figure 1; *p* < 0.05), increased their food intake (1,427.9 ± 313.2 g/group), body weight (297.0 ± 54.2 g/rat) and physical activity, and reduced their respiration rate (94.50 ± 10.53 breath/min), heart rate (417.04 ± 11.06 beat/min) as well as fur dullness. Rats treated with PZWH had a liver elasticity similar to those of receiving the ursodeoxycholic acid (Figure 1).

### 3.3 Liver macroscopic and histopathological profiles

**FIGURE 2:**
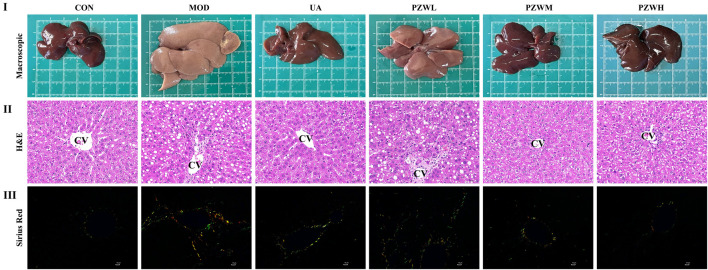
(I) Macroscopic, (II) histopathological (magnification factor: ×400) and (III) sirius red staining study of liver in normal rats, rats with carbon tetrachloride-induced liver fibrosis and those treated with UA and PZW. CV: central vein.

Macroscopic observation of liver of MOD showed low gloss, rough surface texture, severe adhesion between the neighboring lobes and thickened tissues when compared to that of CON (Figure 2I). Histopathological examination indicated that the liver of MOD was characterized by structural disorganization of the hepatic lobules, presence of a large number of steatotic vacuoles of varying sizes in hepatocytes, and hyperplasia of fibrous connective tissues in the portal area, which were segregated into pseudofollicles of varying sizes (Figure 2II). Treatment of these rats with PZW regained their macroscopic appearance and texture in a dose-dependent manner (Figure 2II). The macroscopic structure of liver in rats receiving PZWH was similar to that of treating with ursodeoxycholic acid. PZW significantly improved the morphological structure of the liver with reduced fibrous connective tissue, pseudolobules and inflammatory cell infiltration (Figure 2II). Sirius red staining study revealed that PZW significantly decreased the collagen deposition around the porta hepatis and fibrotic area (Figure 2III). The collagen deposition in liver tissue of rats was similar to that of treating with ursodeoxycholic acid.

### 3.4 Serum biochemical and inflammation/fibrosis marker profiles

**FIGURE 3:**
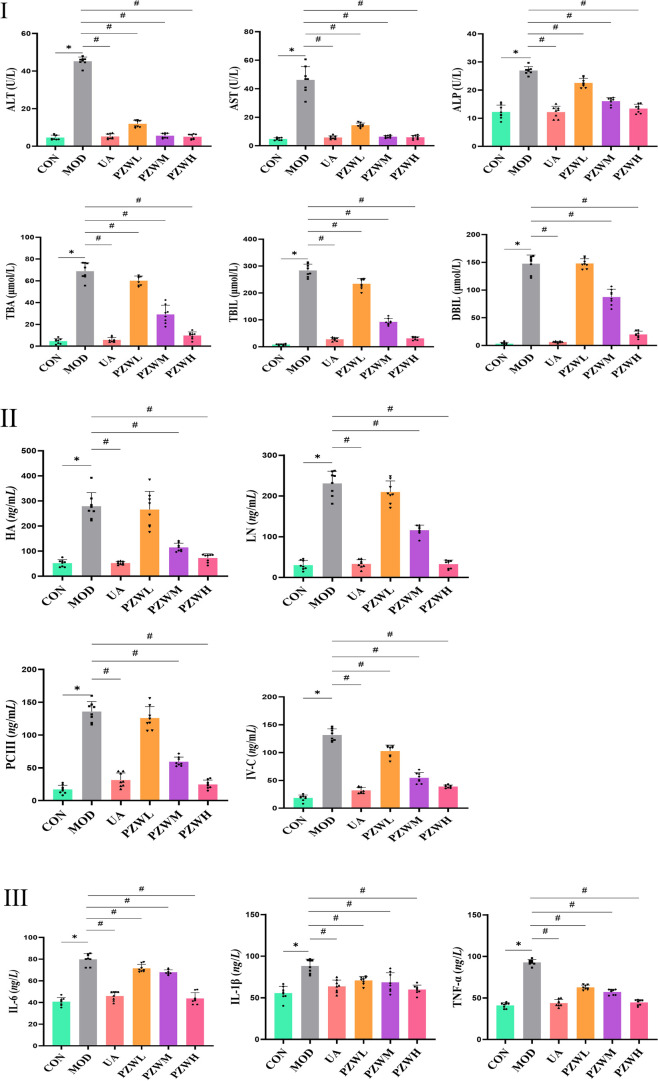
Profiles of serum (I) ALT, AST, ALP, TBA, TBIL and DBIL (liver function indicator); (II) HA, LN, PC-III and IV-C (liver fibrosis indicator); (III) IL-6, IL-1β and TNF-α (liver inflammation indicator) levels in normal rats, rats with carbon tetrachloride-induced liver fibrosis and those treated with UA and PZW. The serum marker level was expressed as mean ± standard deviation. * *p* < 0.05 against CON, # *p* < 0.05 against MOD.

Serum ALT, AST, ALP, TBA, TBIL and DBIL levels were significantly raised in MOD due to carbon tetrachloride-induced hepatic fibrosis as evidenced by hepatic shear wave elastography, macroscopic and histopathological analysis (Figure 3I; *p* < 0.05). PZW significantly reduced the serum ALT, AST, ALP, TBA, TBIL and DBIL levels in MOD as a function of dose (*p* < 0.05). A high PZW dose regained the liver normality as in the case of rats treated with ursodeoxycholic acid (*p* > 0.05). In conjunction with carbon tetrachloride-induced liver fibrosis, the serum levels of IL-6, IL-1β and TNF-α as well as HA, LN, PC-III and IV-C in MOD were significantly elevated against that of CON (Figure 3 II and III; *p* < 0.05). Early in liver injury, inflammation drives disease progression. Multiple immune-inflammatory factors are involved in the regulation of fibrosis, and interleukins are the key components of immune regulation, including IL-6 and IL-1β, which play a pro-fibrotic role. TNF-α, on the other note, regulates the expression of matrix metalloproteinase enzymes and promotes ECM remodeling. The serum IL-1β, IL-6 and TNF-α as well as HA, LN, PC-III and IV-C levels decreased with rats treated with PZW (Figure 3 II and III; *p* < 0.05). PZW ameliorated the carbon tetrachloride-induced liver fibrosis by attenuating the hepatic inflammatory response. At a high PZW dose, the extent of hepatic inflammation was reduced to a comparable degree as rats administered with ursodeoxycholic acid. The recovery extent from liver fibrosis was similar between UA and PZWH groups.

### 3.5 MMP1/TIMP1 balance and collagen deposition

**FIGURE 4:**
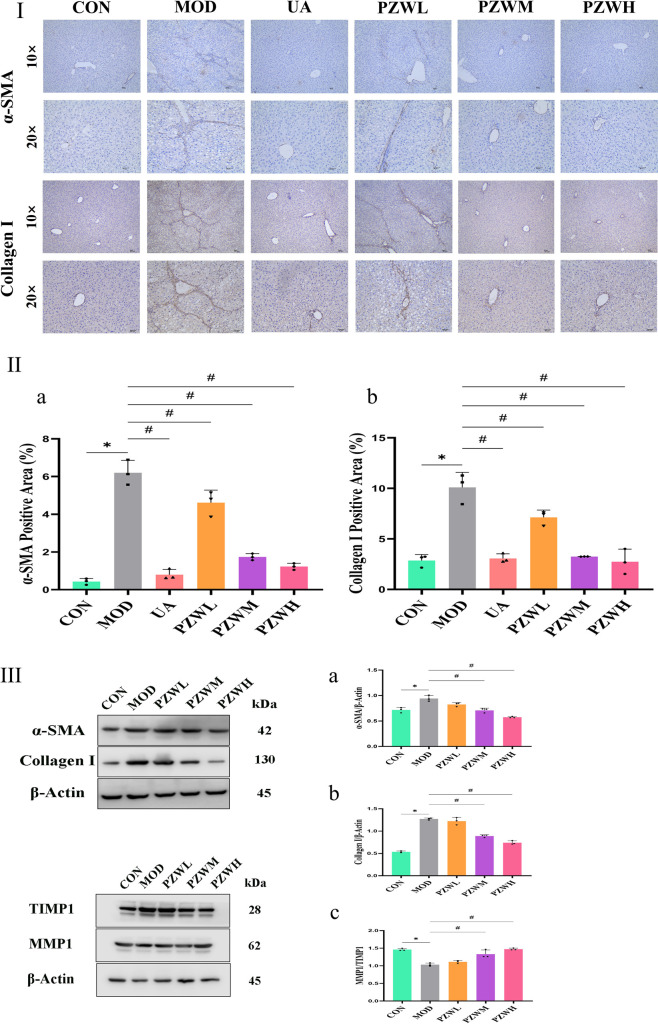
**(Continued).** Immunohistochemistry evaluation of liver tissue in normal rats, rats with carbon tetrachloride-induced liver fibrosis and those treated with UA and PZW: (I) histological representation, (II) percentage of **(a)** α-SMA, **(b)** collagen I; (III) Western blotting evaluation of liver in normal rats, rats with carbon tetrachloride-induced liver fibrosis and those treated with PZW: expression of **(a)** α-SMA, **(b)** collagen I, **(c)** MMP1/TIMP1 ratio. The result was expressed as mean ± standard deviation. * *p* < 0.05 against CON, # *p* < 0.05 against MOD.

The development of liver fibrosis was characterized by hepatic inflammatory responses and excessive collagen deposition which was reflected by enhanced liver elasticity (Figures 1, 3, 4). The latter was further verified by immunohistochemistry and Western blotting evaluation of liver tissue where the expression level of α-SMA and collagen I in CON became higher in MOD (Figure 4). These marker levels decreased when the MOD was treated with PZW (*p* < 0.05). The MMP1/TIMP1 ratio in MOD was lower than that of CON (*p* < 0.05). A comparatively low expression of MMP1 in MOD translated to reduced collagen degradation, while a relatively high TIMP1 expression prevented collagen breakdown. The summative effect was excessive collagen deposition in ECM and the formation of fibrotic liver tissue. Treatment of rats with 120 mg/kg PZW (PZWM) and 240 mg/kg PZW (PZWH) led to an increase in the MMP1/TIMP1 ratio to a level close to CON (Figure 4III). Excessive collagen and ECM were broken down in PZWM and PZWH groups thereby reducing the extent of liver fibrosis.

### 3.6 IL-6/JAK2/STAT3 and TGF-β/Smad expression and signaling pathway

**FIGURE 5:**
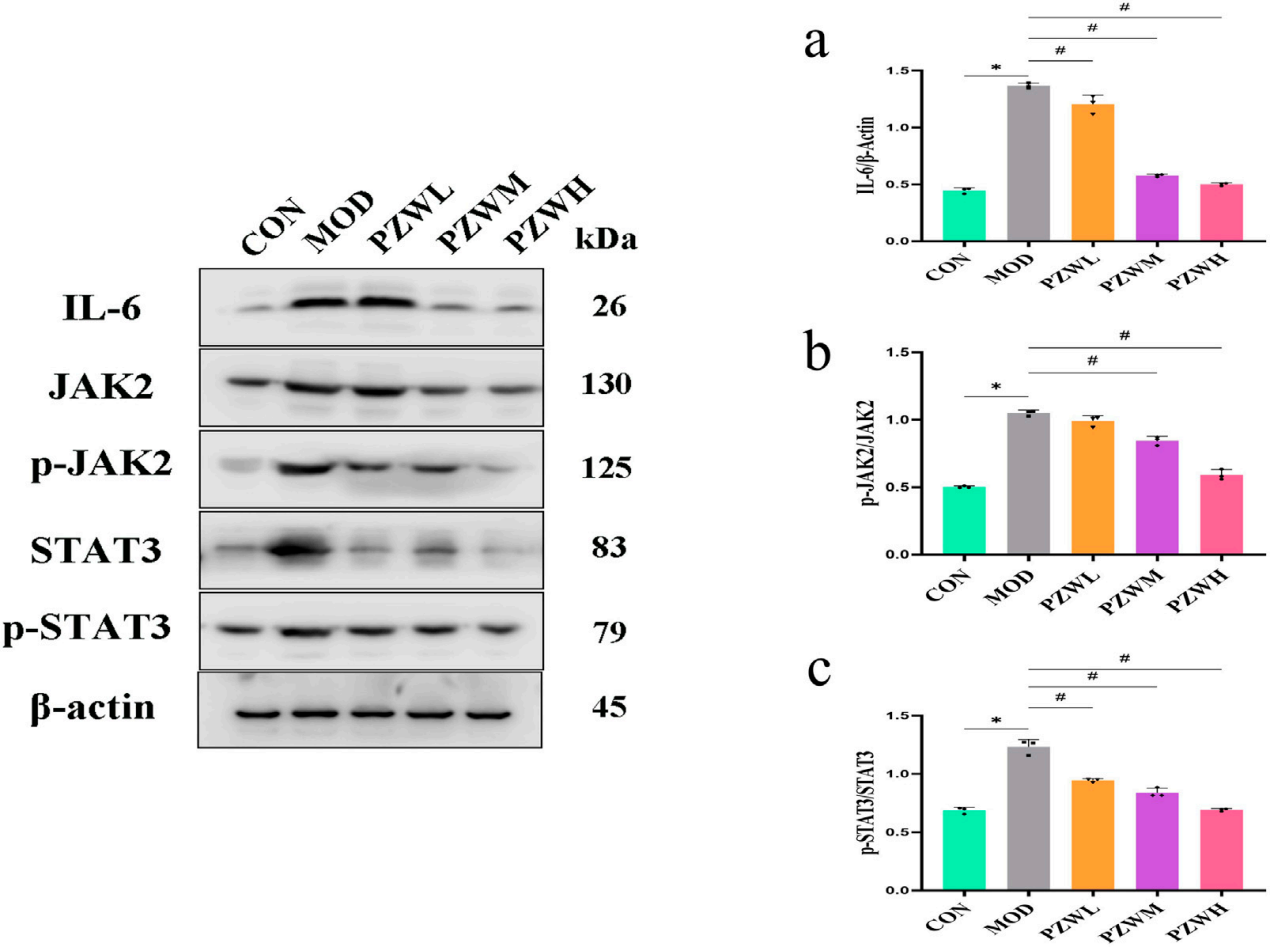
Expression of **(a)** IL-6, **(b)** p-JAK2/JAK2 and **(c)** p-STAT3/STAT3 in normal rats, rats with tetrachloride-induced liver fibrosis and those treated with PZW. The protein expression was expressed as mean ± standard deviation. *p < 0.05 against CON, # *p* < 0.05 against MOD.

**FIGURE 6:**
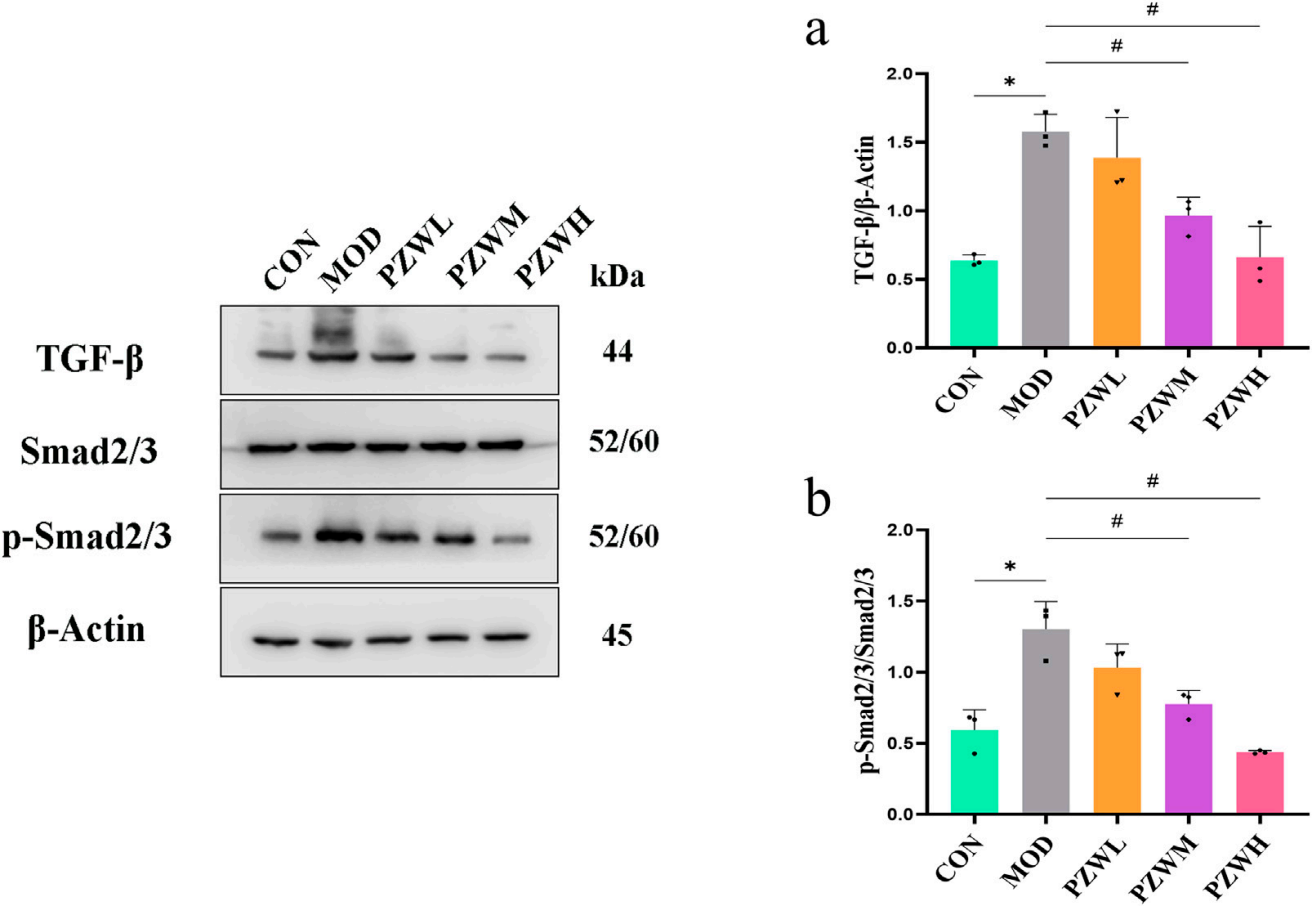
Expression of **(a)** TGF-β and **(b)** p-Smad2/3/Smad2/3 in normal rats, rats with tetrachloride-induced liver fibrosis and those treated with PZW. The protein expression was expressed as mean ± standard deviation. *p < 0.05 against CON, # *p* < 0.05 against MOD.

The expression of IL-6, p-JAK2, p-STAT3, TGF-β and p-Smad2/3 in liver tissue was raised in MOD with CON intoxicated with carbon tetrachloride (Figures 5, 6). Their expression level was reduced in PZWM and PZWH (*p* < 0.05). The JAK2/STAT3 signaling pathway is closely related to the development of hepatic fibrosis, and inhibition of JAK2/STAT3 signaling pathway activity can negate the expression of inflammatory factor and improve hepatic fibrosis. Smad2/3 signaling pathway mediated by TGF-β has been found to be the most important route mediating collagen deposition. Apparently, PZW could mitigate hepatic fibrosis by inhibiting the IL-6/JAK2/STAT3 and TGF-β-Smad2/3 signaling pathways, attenuating collagen deposition and promoting collagen degradation. Correlation analysis suggested that the elasticity value was positively correlated with ALT, AST, ALP, TBA, TBIL, DBIL, IL-6, TNF-α, IL-1β, α-SMA and collagen I (*p* < 0.05). The α-SMA and collagen I was positively correlated with ALT, AST, ALP, TBA, TBIL, DBIL, IL-6, TNF-α, IL-1β, respectively (*p* < 0.05). It is generally recognized that the hepatic stellate cells play an important role in the accumulation and remodeling of ECM into collagen-rich hepatic parenchyma tissue (García-Sáez et al., 2024). Activation and proliferation of hepatic stellate cells involving JAK2/STAT3 pathway is a key link in fibrogenesis and fibrotic liver development. In the course of liver injury, the damaged liver parenchymal cells, hepatic sinusoidal endothelial cells and kupffer cells could release cytokines to activate the hepatic stellate cells, of which TGF-β is one of the key mediators. The activated hepatic stellate cells could secrete a large amount of extracellular matrix to initiate the process of hepatic fibrosis *via* the Smad2/3 signaling pathway, and also autocrine TNF-α, TGF-β and other cytokines to sustain the fibrosis activity. Upregulation of TGF-β/Smads and JAK2/STAT3 pathways has been reported to activate the differentiation of hepatic stellate cells into fibroblasts and this further exacerbates liver fibrosis.

MMPs are secreted by several types of cells, including fibroblasts, neutrophils and macrophages of which it has the ability to degrade the ECM and ameliorate liver fibrosis (Kamio et al., 2010). TIMP1 is produced by hepatic stellate cells, and its overexpression inhibits MMP1 activity thus leading to reduced ECM degradation. PZW negated the downshift of MMP1/TIMP1 ratio and reduced the fibrosis extent of the liver. IL-6/JAK2/STAT3 and TGF-β/Smads are key signaling pathways which induce and sustain fibroblast activity as well as fibrotic tissue remodeling (Chen et al., 2020). PZW ameliorated liver fibrosis, and this effect was accompanied by reduced phosphorylation of JAK2, STAT3 and Smad2/3.

## 4 Discussion

The UHPLC-MS analysis identified six main compounds in PZW: piperine, trehalose, mulberroside F, chebulic acid, gallic acid and hydroxysafflor yellow A. Each of these compounds contributes to PZW’s anti-hepatic fibrotic effect. Piperine is an amide alkaloid available abundantly in fruits and roots of black pepper with a variety of pharmacological properties. It has recently been identified as a promising anti-fibrotic agent against liver and pancreatic fibrosis through altering the TGF-β/Smad pathway (Abdelhamid et al., 2021; Choi et al., 2019; Ma et al., 2017a) and able to inhibit JAK2/STAT signaling pathway in a silicosis mouse model to reduce inflammatory cell infiltration and collagen deposition (Cai et al., 2025). Trehalose (α-D-glucopyranosyl α-D-glucopyranoside), a non-reducing disaccharide, has been shown to protect proteins and cellular membranes from inactivation or denaturation caused by different stress conditions, and protect hepatocytes from palmitate-induced toxicity (Minutoli et al., 2008). It is found to reduce TGF-β1-induced α-SMA and collagen I expression in peritoneal mesothelial cells and conjunctival fibroblasts, indirectly inhibiting the fibrotic response (Miyake et al., 2020; Wu et al., 2020). Mulberroside F has been shown to reduce the secretion of inflammatory cytokines, chemokines and eotaxin-1 (CCL11) as well as reduce the reactive oxygen species (ROS) production which collectively mitigate immune-responses as inferred by the inflamed tracheal epithelial cells (Huang et al., 2023). In asthmatic mice, it reduces pulmonary inflammation and collagen deposition. Chebulic acid, available in *Chebulae* Fructus, displays protective effects against liver damage by oxidative stress both *in vivo* and *in vitro*, and *in vivo* inhibitory effects against hepatic fibrosis (Koo et al., 2016). Gallic acid, a widespread polyphenol in plants, is reported to act as a free radical scavenger showing anti-inflammatory and anti-mutagenic properties that collectively exert hepatoprotective effects (Zhang J. et al., 2023). In both *in vitro* and *in vivo* liver fibrosis model, it suppresses HSC activation by inhibiting TGF-β1/Smad3 pathway thus reduces α-SMA and collagen I expression as well as targets JAK2/STAT3 pathway to regulate cell proliferation and apoptosis (El-Lakkany et al., 2018; Hu et al., 2018; Tang et al., 2020). Hydroxysafflor yellow A, an active principle of *C. tinctorius* L. (Asteraceae; safflower), is extensively employed in traditional Chinese medicine for the treatment of cirrhosis, cerebrovascular and cardiovascular diseases (Liu et al., 2014). It inhibits HSC activation and blocks TGF-β1-regulated fibrotic signaling (Zhang et al., 2011). The summative chemical profiles suggest that PZW is favorable for use in treating liver-related medical conditions. In comparison to Triphala with similar bioactivities, PZW shares similar chebulic acid and contains hydroxysafflor yellow A of which is not identified in Triphala (Bairwa et al., 2025; Long et al., 2023). Triphala’s bioactivities rely on Nrf2 activation and related pathways could be elucidated in the case of PZW (Wei et al., 2021). PZW presents as a more efficacious anti-fibrotic compound when compared to silymarin with weaker MMP1 up-regulatory effects and salvianolic acid B with a high dose requirement ≥300 mg/kg (Kara et al., 2008; Ma et al., 2017b). Overall, the active compounds demonstrate no toxicity at the adopted PZW doses with reference to body weight-specific dose and No-Observed Adverse-Effect Level (Kim et al., 2012; Liu et al., 2004; 2013; Niho et al., 2001; Piyachaturawat et al., 1983).

Liver fibrosis is characterized by excessive ECM deposition, which is tightly regulated by the balance between MMPs and TIMPs (Khurana et al., 2021). In the hepatic fibrosis model group, the MMP1/TIMP1 ratio was significantly decreased thus leading to reduced ECM degradation and increased collagen accumulation. PZW treatment, especially at medium and high doses, restored the MMP1/TIMP1 ratio to near-normal levels. PZW promoted collagen degradation by upregulating MMP1 or downregulating TIMP1, and this was evidently reflected histologically.

*In vivo* analysis confirmed that PZW downregulated the phosphorylation of JAK2, STAT3, and Smad2/3, thereby inhibiting the activation of these pro-fibrotic pathways. The IL-6/JAK2/STAT3 pathway is involved in the activation of hepatic stellate cells (Cao et al., 2019; Handy et al., 2011; Zhang Y. et al., 2023), while the TGF-β/Smad2/3 pathway is the primary mediator of collagen synthesis (Bai et al., 2023; Khongpiroon et al., 2025). By suppressing both pathways, PZW not only reduced hepatic stellate cell activation (evidenced by decreased α-SMA expression) but it also inhibited collagen production (reduced collagen I expression), leading to a comprehensive anti-fibrotic effect.​

Correlation analysis highlighted that liver elasticity (a marker of fibrosis severity) was positively correlated with α-SMA, collagen I and pro-inflammatory cytokines. This suggested that the inhibitory actions of PZW on hepatic inflammation and pro-fibrotic pathways collectively contributed to reduced liver stiffness. The anti-fibrotic effects of PZW were mediated in a dose-dependent manner.

## 5 Conclusion

**FIGURE 7:**
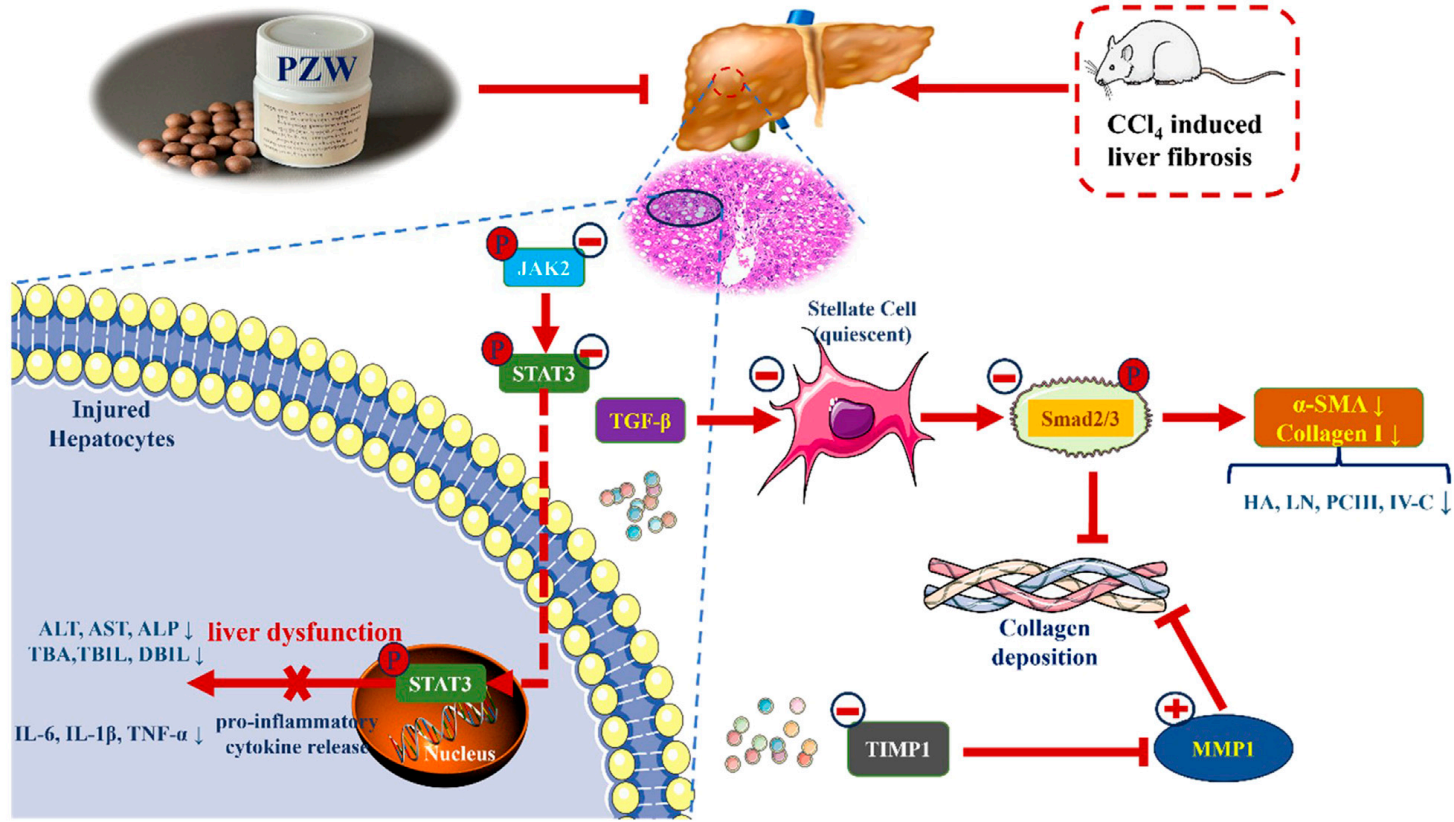
Schematic representation of the mechanisms of anti-hepatic fibrosis action of PZW.

Piperine, trehalose, mulberroside F, chebulic acid, gallic acid and hydroxysafflor yellow A were the main compounds of PZW. They possess anti-inflammatory, anti-oxidant and hepatoprotective activities. PZW alleviated liver fibrosis in a dose-dependent manner with reduced fibrous collagen deposition, pseudolobule formation and inflammatory cell infiltration. The serum IL-1β, IL-6, TNF-α, TBA, TBIL, DBIL, ALT, AST, ALP, HA, LN, PC-III, and IV-C levels decreased with rats treated with PZW leading to reduced liver inflammation and fibrosis and improved overall hepatic health (Figure 7). PZW mitigated hepatic fibrosis, with its effect associated with the inhibition of the IL-6/JAK2/STAT3 and TGF-β/Smad2/3 signaling pathways and through raising the MMP1/TIMP-1 ratio. The extent of liver fibrosis was reduced by reducing collagen deposition and enhancing collagen degradation. To affirm the practical translation of the present findings, further *in vivo* pharmacokinetic study as well as pharmacodynamic studies examining PZW effects as a function of treatment duration, animal gender and species from tissue to cellular/molecular levels are imperative. Human study involving patients is required to translate PZW for clinical application. *In vivo* and clinical responses of healthy non-fibrotic animals and patients to PZW are essential to be elucidated with respect to liver physiology and histology from the perspective of safety and toxicity.

## Data Availability

The original contributions presented in the study are publicly available. The data can be found here: 10.6084/m9.figshare.30496667.

## Glossary

ALP: alkaline phosphatase
ALT: alanine transaminase
AMPK: 5′adenosine monophosphate-activated protein kinase
ANOVA: one-way analysis of variance
AST: aspartate aminotransferase
BCA: bicinchoninic acid
CCl4: carbon tetrachloride
DAB: diaminobenzidine
DBIL: direct bilirubin
ECL: electrochemiluminescence
ECM: extracellular matrix
ELISA: enzyme-linked immunosorbent test
ERK1/2: extracellular signal-regulated kinase 1/2
HA: hyaluronic acid
HE: hematoxylin-eosin
HRP: horseradish peroxidase
HSCs: hepatic stellate cells
IgG: goat anti-rabbit immunoglobulin G
IκB-α: nuclear factor of kappa light polypeptide gene enhancer in B cells inhibitor alpha
IL-1β: interleukin-1β
IL-6: interleukin-6
IV-C: type IV collagen
JAK2: janus kinase 2
JNK: c-Jun N-terminal kinase
LC-MS: liquid chromatograph mass spectrometer
LN: laminin
MAPK: mitogen-activated protein kinase
MCP1: monocyte chemoattractant protein 1
MMP1: matrix metalloproteinase 1
NF-κB: nuclear factor kappa-light-chain-enhancer of activated B cells
PC-III: type III procollagen
PPAR: peroxisome proliferator-activated receptor
PZW: Pa Zhu Wan
ROS: reactive oxygen species
Smad2/3: mother against decapentaplegic homolog 2/3
Smad7: mother against decapentaplegic homolog 7
SMA: smooth muscle actin
STAT3: signal transducer and activator transcription
TBA: total bile acid
TBIL: total bilirubin
TBST: tris buffered saline with tween 20
TGF-β: transforming growth factor-β
TIMP1: tissue inhibitors of metalloproteinases 1
TNF-α: tumor necrosis factor-α
TOF/MS: triple-time-of -flight mass spectrometry
UA: ursodeoxycholic acid
UHPLC: ultrahigh performance liquid chromatography
