# Supplementary material for: Tibetan medicine Pa Zhu Wan ameliorates carbon tetrachloride-induced liver fibrosis in rats by regulating the TGF-β-Smad2/3 and IL-6/JAK2/STAT3 signaling pathways
Source: Front Pharmacol. 2025 Nov 10;16:1667685. doi: 10.3389/fphar.2025.1667685 (PMC12640923; doi:10.3389/fphar.2025.1667685)
Supplement: Supplementary file 1 [file Supplementaryfile1.docx]

**TABLE S1** Composition of PZW.

| Local name | Botanical name | Common name | Part of plant used |
| --- | --- | --- | --- |
| Bi Ba | *Piper longum L.* | *Fructus Piperis Longi* | Fruit |
| Dou Kou | *Amomum Roxb.* | Cardamom | Fruit |
| Guang Ming Yan | *Halite.* | Bright Salt | Mineral mannite |
| Gui Pi | *Cinnamomum tamala (Bauch.-Ham.) Nees et Eberm.* | Cinnamon | Bark |
| Hong Hua | *Carthamus tinctorius L.* | Saffron | Flower |
| Hu Jiao | *Piper nigrum L.* | Pepper | Fruit |
| Han Shui Shi | *Gypsum Rubrum.* | Crystalline mirabilite | Mineral mannite |
| He Zi | *Terminalia chebula Retz.* | *Chebulae Fructus* | Fruit |
| Mu Xiang | *Auckiandia lappa Decne.* | *Radix Aucklandiae* | Root |
| Shi Liu | *Punica granatum L.* | Pomegranate | Pericarp |
| Ye Jiang | *Zingiber striolatum Diels.* | Zingiberaceae | Root |

**
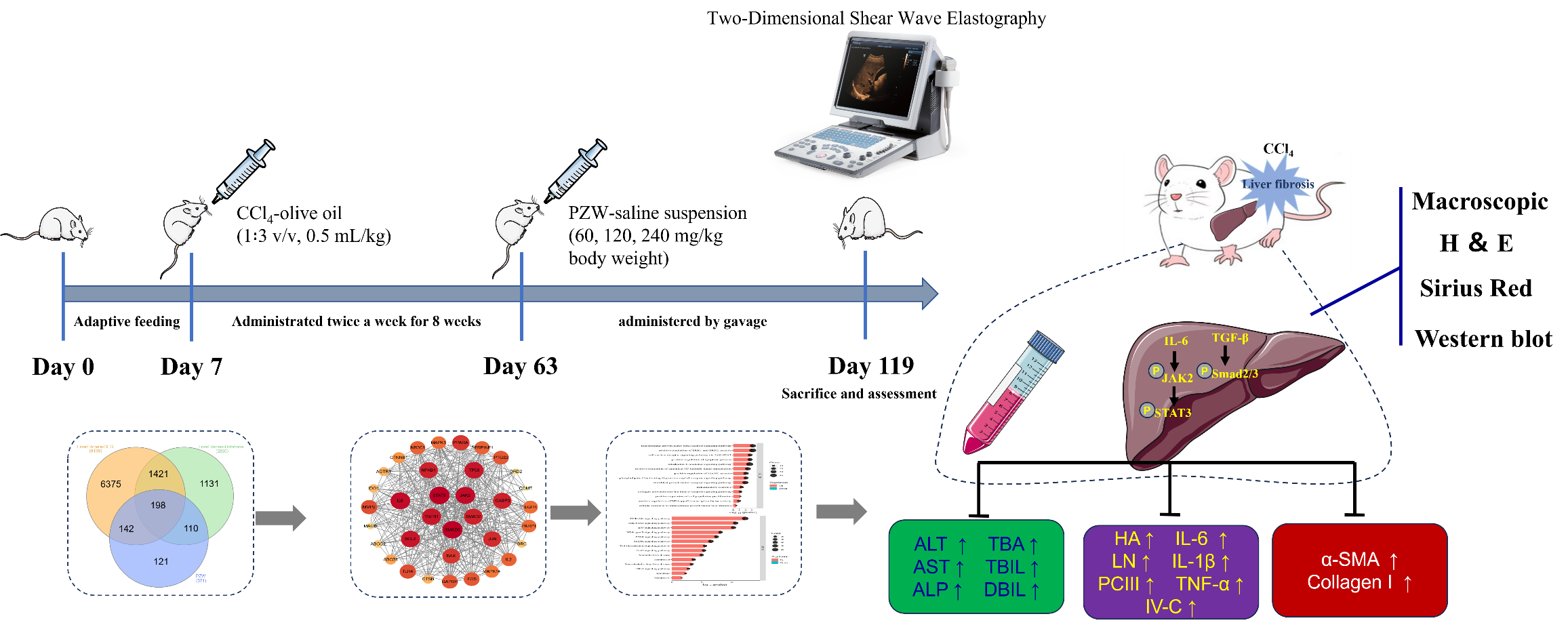
**

**FIGURE S1** Workflow diagram


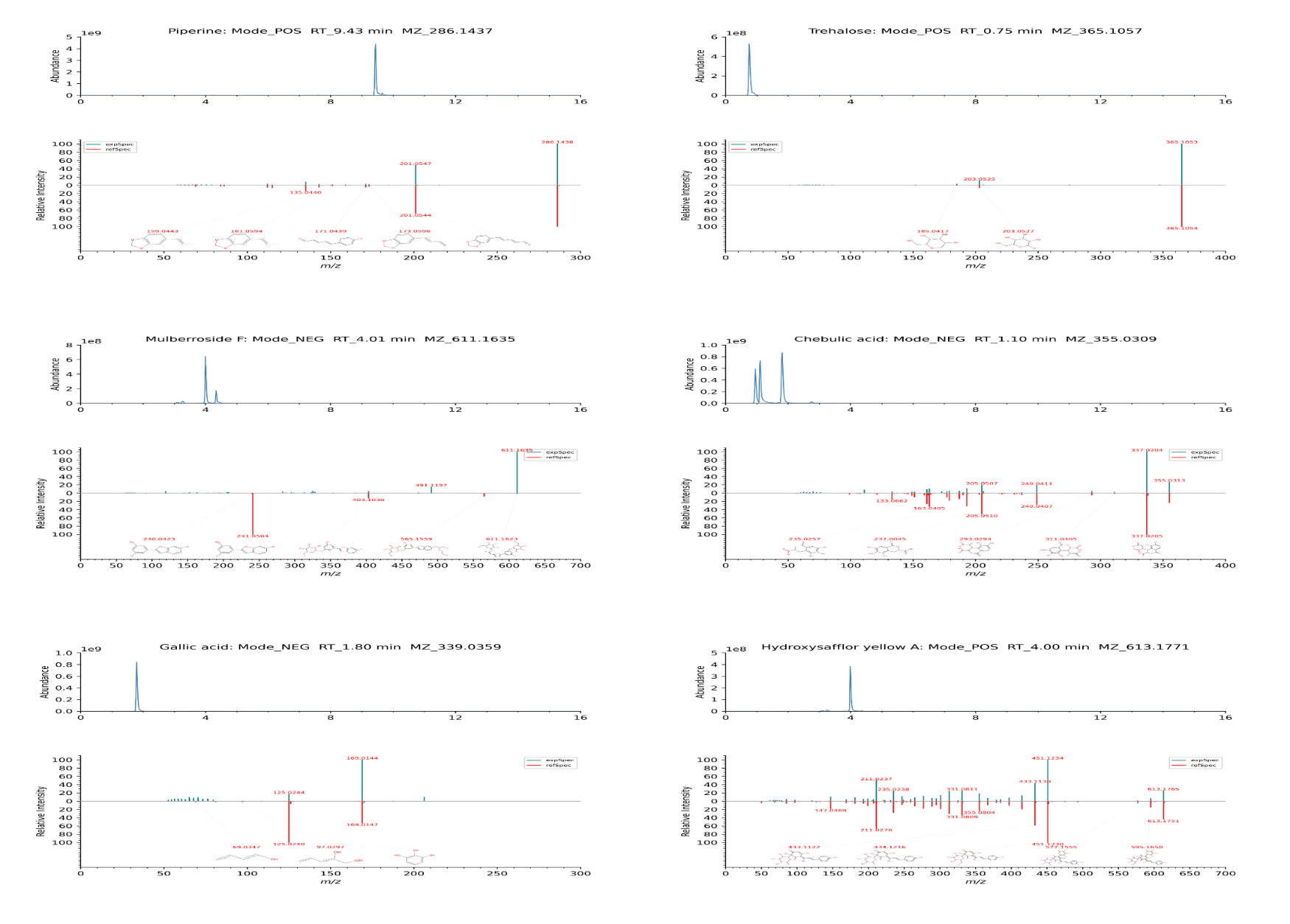


**
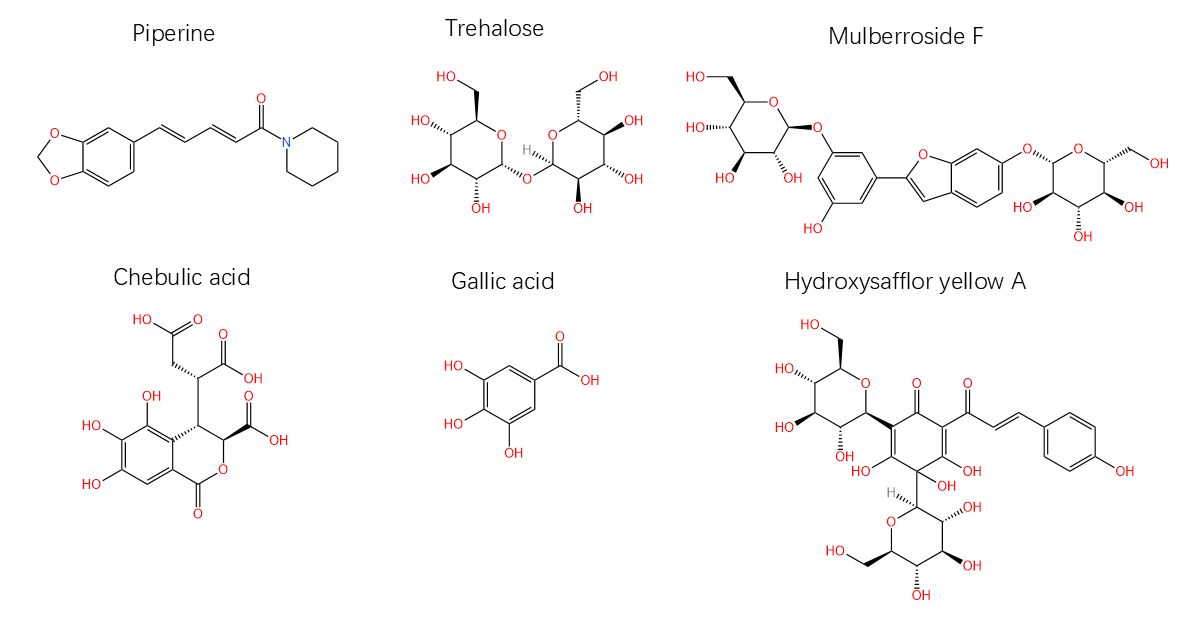
FIGURE S2** Extracted-ion chromatogram plots and chemical structures of the key components of PZW and their MS/MS plots against the standard library LuMet-TCM.
